# Mechanisms of Anti-PD Therapy Resistance in Digestive System Neoplasms

**DOI:** 10.2174/0115748928269276231120103256

**Published:** 2024-01-04

**Authors:** Yuxia Wu, Xiangyan Jiang, Zeyuan Yu, Zongrui Xing, Yong Ma, Huiguo Qing

**Affiliations:** 1 Department of General Surgery, Lanzhou University Second Hospital, Lanzhou, Gansu, China

**Keywords:** Immunotherapy resistance, normalization cancer immunotherapy, PD-1/B7-H1, anti-PD-1/PD-L1 therapy, digestive system neoplasms, pancreatic cancer

## Abstract

Digestive system neoplasms are highly heterogeneous and exhibit complex resistance mechanisms that render anti-programmed cell death protein (PD) therapies poorly effective. The tumor microenvironment (TME) plays a pivotal role in tumor development, apart from supplying energy for tumor proliferation and impeding the body's anti-tumor immune response, the TME actively facilitates tumor progression and immune escape *via* diverse pathways, which include the modulation of heritable gene expression alterations and the intricate interplay with the gut microbiota. In this review, we aim to elucidate the mechanisms underlying drug resistance in digestive tumors, focusing on immune-mediated resistance, microbial crosstalk, metabolism, and epigenetics. We will highlight the unique characteristics of each digestive tumor and emphasize the significance of the tumor immune microenvironment (TIME). Furthermore, we will discuss the current therapeutic strategies that hold promise for combination with cancer immune normalization therapies. This review aims to provide a thorough understanding of the resistance mechanisms in digestive tumors and offer insights into potential therapeutic interventions.

## 1 INTRODUCTION

Malignant tumors of the digestive system are widespread and pose significant health risks with high rates of illness and death. According to the latest statistics from the American Cancer Society, pancreatic cancer (PC) (12%) liver and esophageal cancer (21%) have the lowest survival rates. Additionally, colorectal cancer (CRC) and PC are the second and third most common causes of cancer-related deaths in men and women, respectively [1]. The traditional treatment strategies for gastrointestinal (GI) malignancies include surgical resection, radiation therapy, chemotherapy, and molecular-targeted drug therapy. Despite significant improvements in patient survival, high rates of metastasis and recurrence persist after treatment, resulting in poor overall survival. In recent years, immunotherapy has rapidly developed and gained attention for clinical applications owing to its promising anti-tumor effects. In contrast to conventional treatments, immunotherapy kills cancer cells by activating anti-tumor immunity and specifically targets cancer antigens to prevent normal cells from being attacked [2, 3]. Currently, Immune checkpoint inhibitors (ICIs), primarily PD-1/PD-L1 blockers have shown remarkable success in treating a variety of malignancies, and their efficacy in many clinical trials has advanced the treatment of CRC, gastric cancer (GC), and other GI tumors [4-7]. Anti-PD therapies specifically target the restoration of lost anti-tumor immunity by addressing immune evasion mechanisms in the TME, with the aim to normalize cancer immunity. This presents significant opportunities and challenges for cancer immunotherapy. ICIs, particularly PD-1/PD-L1 antibodies such as nivolumab, have been approved by the United States Food and Drug Administration for the treatment of GI malignancies, including GC, HCC, and CRC (Table **1**). This approval led to significant improvements in the clinical outcomes of patients with GI tract tumors. Numerous on-going clinical trials are assessing the safety and efficacy of anti-PD-1/PD-L1 immuno-therapies for the treatment of GI malignancies [7, 8]. Despite notable achievements of ICIs, their efficacy is limited to a fraction of cancer patients owing to the development of intricate resistance mechanisms [9, 10]. Therefore, gaining a comprehensive understanding of resistance mechanisms to anti-PD therapy is crucial for advancing cancer immunotherapy of the digestive system.

## 2 MECHANISM OF ACTION OF ANTI-PD THERAPY

Previous studies demonstrated that the immune system plays a paradoxical role in limiting and promoting tumorigenesis and cancer progression. This phenomenon is referred to as cancer immune editing and occurs through a complex process involving three phases: elimination, homeostasis, and escape [11]. During the elimination phase, the innate and adaptive immune systems collaborate to identify and eradicate tumors before they become clinically detectable. In the subsequent equilibrium phase, cellular immunogenicity is edited by the adaptive immune system and tumor growth is restricted. In the final escape phase, tumor growth is no longer limited by immunosuppression and/or the activation of immune destruction pathways [12, 13]. To achieve optimal tumor elimination, a multi-faceted approach is necessary. This involves promoting immune activation and T-cell initiation based on T cells while concurrently inhibiting immunosuppressive signals within the TME. Furthermore, it is essential to sustain the presence of T cells within tumor tissues to ensure continued immune surveillance and effective targeting of tumor cells [14, 15]. The primary function of T cells is based on two-step process: activation and post-initiation. First, the T-cell receptor (TCR), which is unique to each T cell, is activated by a specific peptide antigen (Ag) presented by the major histocompatibility complex (MHC) on antigen-presenting cells (APCs). Second, T cells are activated through the co-stimulation of receptors by coupling their ligands B7-1/2 to dendritic cells (DCs). DCs take up and process tumor-specific antigens released by tumor cells and present them to naïve T cells *via* the MHC, thereby triggering T cell initiation and activation through co-stimulatory signals [16, 17]. Notably, during the activation of naïve T cells, PD-1 can be upregulated upon TCR activation [18], thereby activating the PD-1/PD-L1 pathway and leading to the inhibition of the PI3K/AKt/mTOR downstream signaling pathway. This, in turn, results in the suppression of T cell proliferation and differentiation, and leading to its dysfunction and apoptosis [16, 19], tumor cells exploit this mechanism to evade the immune system, thereby contributing to tumor cell relapse and metastasis. It is through the pathway of PD-1/PD-L1 inhibition that anti-PD therapy works [20].

## 3 MECHANISMS OF RESISTANCE TO ANTI-PD THERAPY

TME is a complex composition of various cellular components, including fibroblasts, endothelial cells, immune cells, adipocytes, and extracellular matrix (ECM) [21]. Evidence suggests that the TME is a critical site for the development of drug resistance in malignant tumours [22]. Apart from supplying energy for tumor proliferation and impeding the body's anti-tumor immune response, the TME actively facilitates tumor progression and immune escape *via* diverse pathways, which include the modulation of heritable gene expression alterations and the intricate interplay with the gut microbiota. Therefore, a comprehensive understanding of the TIME is essential for developing future immunotherapy therapies for digestive tumors. Most evidence concerning resistance to anti-PD therapy relates to immune-mediated pathways. Consequently, this review primarily focuses on the immune evasion mechanisms of cancers, particularly in the TME. Additionally, we discuss the impact of metabolism, microbiota, and epigenetics on the response of gastrointestinal tumors to anti-PD therapy.

## 4 IMMUNE-MEDIATED MECHANISMS OF DRUG RESISTANCE

### 4.1 Lack of PD-L1 Expression in the TME

**Fig. (1):**
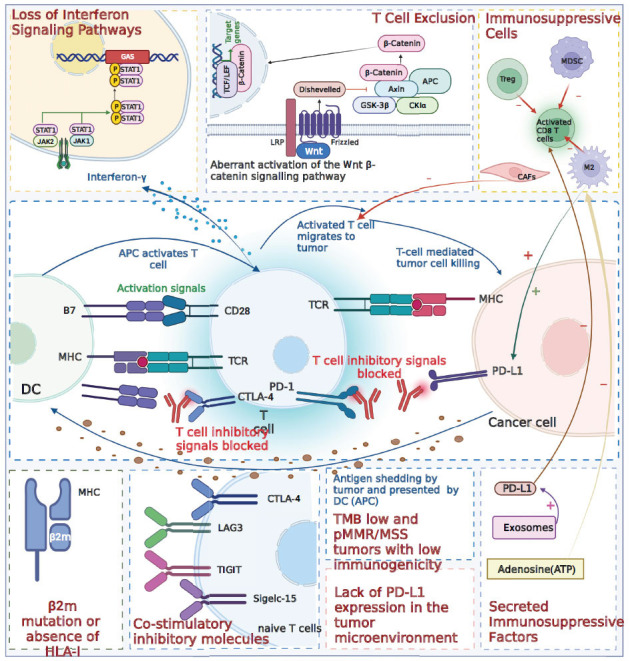
Immune-mediated mechanisms of drug resistance.

The primary objective of anti-PD therapy is to disrupt the PD-1/B7-H1 pathway by administering monoclonal antibodies against B1-H7 or PD-1 in the TME [20]. However, tumors that lack expression of PD1 or PDL1 are classified as “target deficient”, which represents a significant challenge to effective treatment. Prior investigations have categorized tumors into four subtypes based on the presence or absence of PDL1 expression and tumor-infiltrating lymphocytes (TILs) within the TME. Notably, both type I and type III tumors lack PD-L1 expression, and 60-85% of solid tumors display target-deficient resistance mechanisms [23]. Consequently, the absence of immune checkpoint expression within the TME plays a vital role in the development of resistance to anti-PD therapy (Fig. **1**).

### 4.2 T Cell Exclusion

PD-1 is primarily expressed in lymphocytes, specifically in T cells. The lack of T cells within the TME signifies the presence of an additional resistance mechanism [22]. Tumors are categorized as either “hot” or “cold” based on the extent of T-cell infiltration, with “hot” tumors exhibiting and “cold” tumors lacking both inflammation and infiltration [24]. Among tumors in the digestive system, GCs are predominantly categorized as “hot” tumors, whereas colorectal, pancreatic [25], and bile duct [26] cancers are primarily classified as “cold” tumors. Oncogenic pathways operating within cancer cells frequently facilitate evasion of the anti-tumor immune response by inducing T cell exhaustion within the TME [27]. Notably, Phosphatase and tensin homolog (PTEN) have been implicated in the promotion of T cell infiltration in tumors. Evidence suggests that activating mutations in PIK3CA (phosphatidylinositol-4,5-bisphosphate 3-kinase catalytic subunit alpha) and deletion of PTEN drive the accumulation of myeloid-derived suppressor cells (MDSCs) in CRC through GM-CSF signaling, thereby facilitating immune evasion. A clinical study demonstrated that gastrointestinal patients with deficient DNA mismatch repair/microsatellite instability-high (dMMR/MSI-H) and PTEN deletion displayed insensitivity to ICIs, reduced levels of CD8+ T cells, and increased presence of M2 tumor-associated macrophages (TAMs) [28]. Additionally, aberrant activation of the WNT-β-catenin pathway has been associated with the inadequate infiltration of T cells into tumors. Dysregulated activation of Wnt/β-catenin signaling contributes to T cell exhaustion within the CRC tumor microenvironment, resulting in a non-T-cell inflammatory phenotype [29]. Similarly, in hepatocellular carcinoma (HCC), aberrant WNT/β-catenin signaling inhibits dendritic cell recruitment and diminishes T cell infiltration [30]. Furthermore, the expression of inhibitory co-stimulatory molecules adversely affects immune cell function and secretion of factors, leading to T cell exhaustion (Fig. **1**) [31].

### 4.3 Loss of Interferon Signaling Pathways

Interferons play a crucial role in orchestrating anti-tumor immune responses. Among them, IFN-γ is predominantly secreted by Teffs and engages the JAK/STAT pathway in tumor cells. This engagement not only exerts anti-proliferative effects on cancer cells, but also stimulates the expression of MHC class I molecules and chemokines that attract T cells. Paradoxically, IFN-γ also facilitates immune escape by inducing the expression of PD-L1 [32, 33]. In most gastrointestinal cell lines, the MAPK and PI3K-AKT pathways have been implicated in the activation of IFN-γ. In CRC, it has been further elucidated that IFN-γ triggers PD-L1 expression through the JAK1/STAT2 signaling pathway [34]. In HCC, excessive and sustained activation of IFN-γ leads to the downregulation of IFN-γ receptors in HCC cells, ultimately promoting immune escape by diminishing their sensitivity to IFN-γ. Furthermore, IFN-γ induces the production of IL-10 or vascular endothelial growth factor (VEGF) and upregulates PD-L1 expression, fostering the development of acquired drug resistance to ICIs in HCC (Fig. **1**) [35].

### 4.4 Antigen Loss Through Immunoediting

Tumor cells exhibit distinct immunogenicity compared to normal cells, owing to genetic mutations that lead to the expression of specific antigenic phenotypes [35]. Dysfunction of mismatch repair proteins (MMR) leads to the accumulation of errors in DNA microsatellite regions, resulting in microsatellite instability (MSI) [36]. The extent of genome-wide microsatellite instability and subsequent tumor mutational load significantly impacts the effectiveness of anti-PD-1 therapy [37]. Tumors displaying MSI-H are particularly immunogenic because of the abundance of neoantigens [38, 39]. Tumor mutational load (TMB) correlates with the number of neoantigens and reflects the frequency of neoantigen mutations. Higher mutation levels trigger the expression of immunogenic and tumor-specific neoantigens, eliciting potent anti-tumor immune responses [40]. Tumors with low TMB exhibit reduced immunogenicity, potentially leading to resistance to immunotherapy [41]. For instance, pancreatic cancer, which is characterized by a low mutational load, demonstrates poor responsiveness to checkpoint inhibitors [42]. Conversely, CRC with MSI exhibits a high number of tumor-associated neoantigens, making it suitable for ICI therapy [29]. Studies have demonstrated that CRC with dMMR/MSI-H displays elevated tumor mutational load and increased density of TILs compared to proficient mismatch repair (pMMR)/microsatellite stable (MSS) CRC [43]. In conclusion, gastrointestinal tumors, specifically the dMMR/MSI-H and TMB-H subtypes, benefit significantly from anti-PD-1/PD-L1 inhibitors, whereas the effectiveness of these inhibitors against the pMMR/MSS and low mutational load subtypes remains unclear. This uncertainty may be attributed to the loss of tumor-specific antigens. Furthermore, tumor-specific promoters diminish tumor antigenicity by depleting immunogenic peptides, facilitating cancer immune editing, and enabling tumor evasion of the host immune system (Fig. **1**) [44].

### 4.5 Defects in Tumor Antigen Presentation

Tumor cells may exhibit deficiencies in antigen processing and presentation mechanisms, hindering the ability of the immune system to detect novel antigens within cancer cells. Human leukocyte antigen (HLA)-I plays a critical role in presenting processed antigens to T cells, facilitated by β2-microglobulin (β2m) to enhance the stability and peptide loading of MHC molecules on the cell surface.β2m mutations have been identified as a significant cause of HLA-I loss in tumor cells [45]. HLA-I deletion and/or β2m mutations are frequently observed in dMMR/MSI-H CRC with high TIL infiltration, which is attributed to immune selection [29, 46]. However, the precise role of β2m mutations in mediating resistance to ICI remains unclear. For instance, dMMR CRC often experiences loss of HLA-I-mediated antigen presentation due to silencing of HLA-I genes, inactivating β2m mutations, or due to other defects in the antigen processing machinery [47-49]. In pancreatic cancer, inadequate antigen presentation hampers immune surveillance and facilitates the evasion of effector T cells, resulting in reduced tumor antigenicity and a poor response to ICI therapy [50, 51]. Studies have demonstrated that MSI-H tumors frequently carry mutations in β2m and HLA-ABC genes, leading to defective antigen presentation and subsequent immune evasion. These mechanisms may involve but are not limited to, diminished responsiveness to IFN-γ signaling due to defective mutations in genes associated with the JAK/STAT signaling pathway, alterations in the metabolic environment, and increased recruitment of suppressor immune cells facilitated by enhanced expression of relevant chemokines (Fig. **1**) [52].

### 4.6 Immunosuppressive Cells

TAMs, regulatory T cells (Tregs), and MDSCs contribute to immunosuppression within the TME through diverse mechanisms (Fig. **1**).

TAMs are crucial components of the TME and influence tumor metastasis and invasion. They can be categorized into two phenotypes: M1 and M2, with M2 macrophages being the predominant TAM phenotype. M2 macrophages play a multifaceted role in tumor progression, angiogenesis, metastasis, and invasion by secreting growth factors and cytokines. Moreover, they can suppress T cell metabolism and function by releasing immunosuppressive cytokines [53]. Macrophages can modulate PD-L1 expression *via* various cytokine-mediated signaling pathways. In colorectal cancer, TAMs directly express PD-L1 under the influence of the TME, leading to CD8+T cell inactivation and apoptosis through the binding of PD-1 to CD8+T cell surfaces [54]. In pancreatic cancer, the binding of TGF-β to its receptor upregulates PD-L1 expression in tumor cells and TAMs through the AKT/NF kB or AKT/β-catenin pathways [55]. TNF-α secreted by TAMs significantly enhances PD-L1 expression [56]. Furthermore, HCC [57], cholangiocarcinoma (CHOL) [58], and GC [59], macrophages have been shown to enhance PD-L1 expression through various cytokinemediated signaling pathways [60].

Tregs represent a highly immunosuppressive subset of CD4+T cells that rely on the expression and activity of the transcriptional regulator forkhead box P3 (FOXP3) [61]. The infiltration of Tregs has been linked to unfavorable clinical outcomes across various cancer types [62]. Previous research also has emphasized the potential significance of targeting vulnerabilities within Tregs as a means to enhance anti-tumour immune responses [63, 64].

MDSCs are immunosuppressive and immature myeloid cell populations that display phenotypic similarities with neutrophils and monocytes. MDSCs influence both innate and adaptive immune responses through hyperactive mechanisms [65]. In colon cancer, MDSCs impede the binding of specific tumor-associated peptides to tumor cell associated MHCs, consequently conferring resistance to antigen-specific cytotoxic T lymphocytes (CTLs) [66]. Additionally, MDSCs suppress the cytotoxic activity of CD8+T cells [67]. Clinical investigations have demonstrated an association between elevated MDSC levels and diminished survival rates in patients with CRC [68].

Cancer-associated fibroblasts (CAFs) are the predominant cellular components in the stromal compartment of pancreatic ductal adenocarcinoma (PDAC) and intrahepatic cholangiocarcinoma (ICC) [69]. These activated CAFs contribute significantly to tumor growth and survival by forming a dense stromal network in PDAC [70]. CAFs exert a restrictive effect on the infiltration of immune cells into the tumor microenvironment by releasing an abundant collagen network. These collagen-rich environments impede T cell proliferation and migration, particularly within regions exhibiting heightened collagen deposition [71]. Importantly, CAFs have demonstrated the capacity to upregulate immune checkpoint molecules on CD4+T cells and CTLs, thereby compromising the immune function of these T cell subsets [72]. Taken together, these findings underscore the substantial role of CAFs as key contributors to immune evasion in cancer cells (Fig. **1**).

### 4.7 Coinhibitory Molecules in the TME

The expression of co-suppressor molecules within the TME represents a fundamental mechanism underlying tumor immune evasion, resulting in the suppression and dysfunction of effector T cells through interactions with their corresponding ligands [73]. Prominent co-inhibitory molecules associated with T cell depletion include PD1, T-cell immunoglobulin and mucin-domain containing-3 (TIM3), cytotoxic T-lymphocyte antigen 4 (CTLA-4), T-cell immunoglobulin and ITIM domain (TIGIT), CD160, lymphocyte-activating 3 (LAG3), and CD244 [74]. Experimental evidence has demonstrated that LAG3 and TIGIT expression are upregulated in mouse HCC cells upon PD1 administration. Notably, TIGIT mediates resistance to PD1 inhibitors in murine models [75]. However, further investigations are warranted to elucidate the potential involvement of other immune checkpoints in drug resistance following anti-PD treatment. Furthermore, Siglec-15 has emerged as a promising target for tumor immunotherapy, particularly in patients who exhibit a limited response to PD-1/PD-L1 inhibitor therapy. Studies have indicated that high expression of Siglec-15 in esophageal squamous carcinoma (ESCC) [76] and PC [77] contributes to tumor immune evasion within the TME, albeit without significant correlation with the PD-1/PD-L1 pathway (Fig. **1**) [78].

### 4.8 Secreted Immunosuppressive Factors

PD-L1, a potent immunosuppressive molecule, inactivates tumor-specific T cells by binding to the inhibitory receptor PD-1 [79]. Adenosine, also a significant contributor to tumor-mediated immunity, exhibits strong immunosuppressive properties. Anti-cancer therapies elicit the release of elevated levels of ATP into the extracellular space, triggering both innate and adaptive immune responses [80]. Then, extracellular ATP undergoes sequential conversion to its metabolites ADP and AMP *via* the action of CD39, ultimately leading to the production of adenosine through the activity of CD73. Adenosine suppresses the function of anti-tumor immune cells while concurrently promoting the activity of regulatory immune cells such as MDSCs and Tregs [81]. In addition to adenosine, other immunosuppressive molecules are present within the TME. For instance, the cancer cell-derived exosome induces the failure of NK cells, leading to resistance to anti-PD1 therapy in HCC [82]. Tumor-derived exosomes facilitate the upregulation of PD-L1-expressing macrophages, thereby promoting immunosuppression and disease progression in ICC [83] and other malignancies (Fig. **1**).

## 5 MICROBIOME

**Fig. (2):**
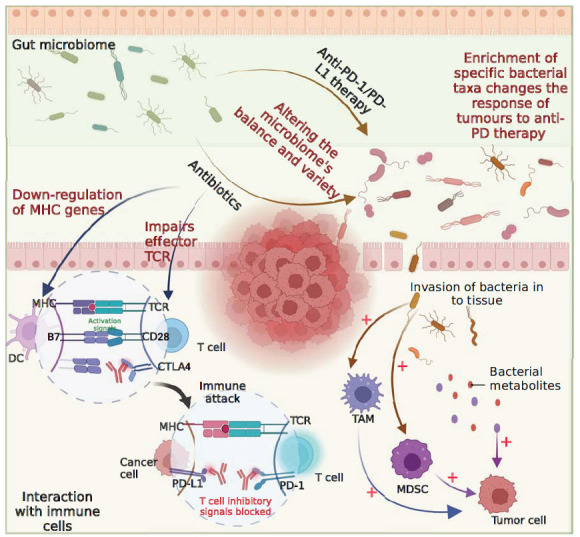
Mechanisms of drug resistance associated with microbiota.

Gut microorganisms play crucial roles in human health by contributing to nutritional metabolism, immune regulation, and protection. Disruption of gut commensal microbiota in mice has been shown to affect the response of tumors to immunotherapy [84-86]. Such gut microbe-dependent therapeutic responsiveness has also been observed in immunotherapeutic interventions cancer patients, indicating that microbes can influence tumorigenesis, progression, and therapeutic efficacy. Additionally, commensal microbiota has been found to affect the therapeutic activity, such as anti-PD-1/PD-L1 monoclonal antibodies (mAbs) (Fig. **2**) [85].

### 5.1 Secreted Immunosuppressive Factors

Research has demonstrated a relationship between the composition of the gut microbiome and the response to anti-PD therapy in gastrointestinal tumors and hepatobiliary cancers [87, 88]. The diversity and composition of the gut microbiome differed significantly between the patients who responded to immunotherapy and those who did not. Studies have revealed that patients with gastrointestinal tract tumors, such as colorectal, esophageal, and GC, who exhibit elevated levels of Prevotella/Bacteroides respond more favorably to anti-PD-1/PD-L1 therapy. Moreover, intestinal bacteria capable of producing short-chain fatty acids, including Eubacterium, Lactobacillus, and Streptococcus, have also been found to be positively associated with anti-PD-1/PD-L1 responses across various GI cancer types [87]. In previous studies examining the gut microbiome of patients with HCC treated with anti-PD-1 immunotherapy, responders and non-responders showed different microbial flora enrichment [88]. In patients with advanced hepatobiliary cancer, a higher abundance of Lachnospiraceae bacterium-GAM79 and Alistipes spp. Marseille-P5997 is associated with longer progression-free survival (PFS) and overall survival (OS) compared to patients with a lower abundance of these microbes [89]. These findings indicate that the enrichment of certain bacterial taxa in the gut microbiota may modify the microbial ecosystem, leading to changes in the tumor response to anti-PD treatment and, consequently, drug resistance.

### 5.2 Abundance and Diversity of Microbial Flora

Derosa *et al.* reported that the use of antibiotics (ATB) within 30 days of initiating ICI therapy in patients with RCC and NSCLC had an adverse impact on both PFS and OS [90]. Recently, a study revealed that the use of antibiotics was significantly associated with poor OS and PFS in cancer patients receiving ICI immunotherapy, especially for those who used antibiotics before the commencement of ICI treatment for tumors such as esophageal cancer (EC) [91]. The probable mechanism underlying the negative impact of antibiotics on ICI action may be the direct bacteriostatic/bactericidal effects of antibiotics that can disrupt the host microbial ecosystem and trigger an alternative microbiome status, resulting in reduced responsiveness to ICI by the down-regulation of MHC class I/II genes and impairment of effector T cell responses. Furthermore, it can alter the pool of microbial-associated molecular patterns (MAMPS) and adversely affect anti-tumour immune responses [92].

### 5.3 Antibiotic Effects

A previous investigation revealed that non-methylated CpG oligonucleotides present in bacterial DNA amplify the anti-tumour immune response of CD8+ T cells by downregulating PD-1 expression through the IL-12 pathway. This suggests that gut bacterial species positively associated with PD-1 and PD-L1 blocking therapies could release components that directly downregulate PD-1 or PD-L1 expression [93, 94]. In MSS CRC, myeloid cells that sense bacteria through toll-like receptors induce the secretion of calcineurin and IL-6, resulting in the expression of the co-inhibitory molecules B7H3 and B7H4 by tumor cells, thereby suppressing the anti-tumour response of CD8+T cells [95, 96]. Furthermore, the symbiotic Fusobacterium nucleatum selectively increases immunosuppressive MDSCs to impede the host anticancer immune response [97]. In PDAC, Lactobacillus activates TAMs and facilitates tumor growth by metabolizing tryptophan found in food [98].

### 5.4 Effect of Metabolic Products

Gut microbiota can produce metabolites that affect local and systemic anti-tumor immune responses, potentially affecting the efficacy of ICIs. A study revealed the translocation of lipoteichoic acid (LTA), a gram-positive gut microbial component, to the liver in obesity-induced HCC. LTA interaction with the obesity-induced gut microbial metabolite deoxycholic acid, eventually promotes HCC progression by inhibiting the production of anti-tumor cytokines by hepatic immune cells [99]. Additionally, the CRC tumor microenvironment has increased metabolic waste due to altered metabolism and proximity to the microbiota, leading to high ammonia levels that induce T cell metabolic reprogramming, increased depletion, and reduced proliferation [100]. Other proposed mechanisms of drug resistance include metabolic substances that increase IFN-γ production by T cells [101], bacterial translocation from the gut to the TME [102], cross-reactivity between cancer antigens and microbial peptides [103], and the direct presentation of bacterial peptides by cancer cells [104]. In conclusion, bacteria can disrupt immune homeostasis *via* various mechanisms, which require further investigation.

In summary, microorganisms promote tumor progression and immunotherapy resistance. Furthermore, there is evidence suggesting that the dietary habits and nutritional status of patients can influence tumor progression and immunotherapy responsiveness [105]. Dietary fiber and probiotics, which modulate the gut microbiome, correlate with varied ICB outcomes [106]. Notably, compounds like short-chain fatty acids (SCFAs) and polyunsaturated fatty acids (PUFAs) demonstrate potential in augmenting immunotherapy efficacy [107].

## 6 METABOLISM

### 6.1 Metabolic Reprogramming

**Fig. (3):**
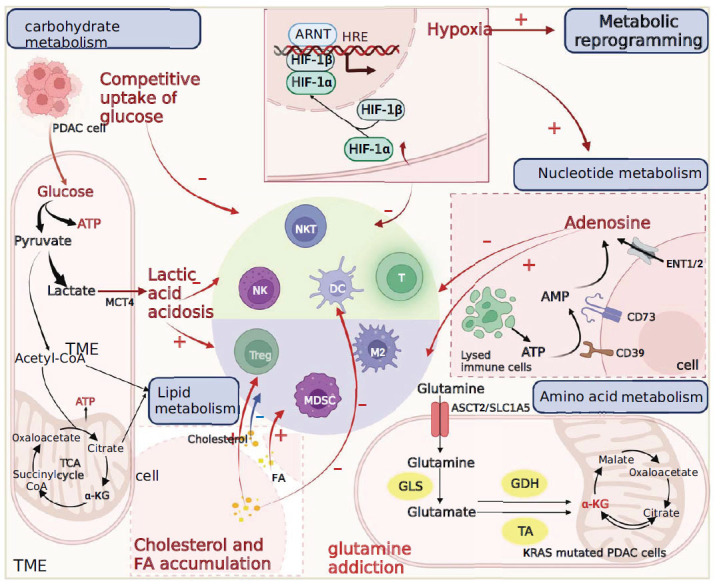
Mechanisms of drug resistance associated with metabolism.

Metabolic reprogramming is a key mechanism underlying tumor immune escape [108], whereby tumor cells exhibit heightened demand for metabolite uptake, translocation, and utilization compared to normal cells. Consequently, proliferating cancer cells within the TIME engage in nutrient competition with immune cells, impairing immune cell function and compromising anti-tumor immunity [109]. Moreover, the altered metabolism of glucose, lipids, and amino acids (AA) leads to the accumulation of metabolites that intricately regulate immune responses through multiple pathways (Fig. **3**) [110].

### 6.2 Carbohydrate Metabolism

Cancer cells exhibit a heightened preference for glycolytic pathways, known as the Warburg effect, to sustain their distinct functional demands within the TME [111]. This metabolic alteration creates a competitive environment for glucose uptake, leading to decreased glucose levels in the TME. Consequently, T-cell metabolism is affected, resulting in impaired cytokine production and reduced T cell responsivenes [112]. The impaired glycolytic capacity of T cells eventually promotes the differentiation of Treg cells [113]. Additionally, glycolysis-derived metabolic intermediates play a pivotal role in macromolecular biosynthesis, conferring a growth advantage to tumor cells under nutrient-restricted conditions. In HCC, the upregulated glycolytic enzyme PFKFB3 induces PD-L1 expression in monocytes *via* NF-κB activation, enabling tumor immune evasion and disease progression [114]. Increased glycolysis in the MSI CRC is also associated with immunosuppression [115]. Furthermore, excessive lactate accumulation resulting from heightened glycolysis facilitated by MCT4 [116] leads to lactic acidosis within the TME. Elevated lactate levels not only impair the anti-tumor effects of immune cells but also promote the expansion of immunosuppressive cells [117, 118]. Moreover, lactate accumulation in tumors regulates cancer cell metabolism and survival, in PC, it also regulates immune escape [119]. Recent findings indicate that Tregs in the acidic TME of highly glycolytic tumors, including MYC-amplified and liver tumors, exhibit increased PD-1 expression. Consequently, the PD-1 blockade amplifies the activity of PD-1+Treg cells, leading to immunotherapy resistance [120]. In conclusion, the aforementioned studies demonstrate that the accumulation of glycolysis and its metabolites, along with the resulting acidosis in the TME, promotes tumor immune escape. Consequently, these immunosuppressive processes contribute to immune escape and the development of tolerance to immunotherapy.

Moreover, emerging research indicates that despite the prevalent reliance on glycolysis as the primary energy source in cancer, there is increasing evidence of mitochondria reprogramming in proliferating cancer cells to meet the elevated energy requirements associated with cell division, migration and invasion [121]. Notably, there is pronounced upregulation of mitochondrial respiratory complex I, both at the protein and transcriptome levels in PDAC. This indicates that cancer cells, including those in PDAC, maintain the coexistence of glycolysis and mitochondrial respiration *via* oxidative phosphorylation as the two pathways for ATP generation [122].

### 6.3 Lipid Metabolism

To meet the energy requirements of tumor cells, lipoprotein lipase secreted by tumor and stromal cells triggers the activation of adipocytes, resulting in the hydrolysis of stored triglycerides and the release of fatty acids (FAs), which are subsequently taken up by the cells. However, within the TME, lipids play a dual role, capable of influencing both anti-tumor and pro-tumor immune responses [123]. The accumulation of FAs hampers the cytotoxicity of effector T cells and enhances their immunosuppressive effects on regulatory T cells. Additionally, elevated lipid levels promote immune resistance in tumors by impairing the antigen presenting capacity of DCs. Notably, intercellular FAs and cholesterol have divergent effects on immunosuppressive cells, such as TAMs and MDSCs [123].

### 6.4 Amino Acid Metabolism

Amino acids serve as a source of energy during tumor cell proliferation and play an indirect role in the modulation of immune cell function, thereby influencing the tumor immune response. Metabolic reprogramming of amino acid pathways is closely associated with tumorigenesis in various human cancers [124], notably in pancreatic cancer, where KRAS mutations drive dependency on glutamine [125]. Tumor cells often exhibit elevated expression of amino acid transporter proteins [126, 127] that compete with immune cells for amino acid uptake. The insufficient availability of specific amino acids and deficiencies in key enzymes involved in amino acid metabolism impair the function of immune effector cells within the TME, consequently leading to resistance to tumor immunotherapy [128].

### 6.5 Nucleotide Metabolism

Altered nucleotide metabolism in cancer cells facilitates immune evasion *via* diverse mechanisms [129]. Adenosine, a purine nucleotide, accumulation within the hypoxic TME [130, 131] and its subsequent interaction with cell surface adenosine receptors modulate signaling pathways in various immune cell subsets. The necrolysis of apoptotic regulatory T cells generates elevated levels of extracellular ATP, which are subsequently converted to adenosine by the cell surface enzymes CD39 and CD73 [132]. These enzymes, upregulated in human cancers, are associated with unfavorable prognoses [133, 134]. Adenosine also can be directly exported from cancer cells *via* equilibrative nucleoside transporter 1 or 2 (ENT1/2). The presence of extracellular adenosine inhibits the antitumor activity of T cells, NK cells, and DCs. Additionally, it fosters the immunosuppressive activity of TAMs and MDSCs, thereby facilitating immune evasion by cancer cells [135, 136].

## 7 HYPOXIA

Hypoxia is a prevalent characteristic of the TME and is recognized as a crucial factor contributing to tumor immune evasion [137]. In the highly fibrous inflammatory stroma, tumors exhibit increased ECM abundance, leading to the compression of blood vessels and severe hypoxia [138]. Hypoxia-inducible factor (HIF) serves as a key regulator of cellular responses to hypoxia. Upregulation of HIF1α promotes the immunosuppressive activity of MDSCs and TAMs [139] and facilitates the presence of Treg cell within the tumor [140]. Additionally, hypoxia contributes to the establishment of an immunosuppressive TME by promoting the expression of PD-L1 [139] and other inhibitory receptors on tumor cells [141], thereby depleting T cells. Furthermore, hypoxic TME generates the cancer metabolite 2-hydroxyglutarate (2HG) [142]. In the PC, L-2HG hampers the infiltration of CD8+T cells by impeding their proliferation and migration, ultimately resulting in resistance to immunotherapy [143]. Hypoxia is intricately associated with metabolic reprogramming, and low oxygen or hypoxic levels induce significant alterations in cellular metabolism. To adapt to hypoxic conditions, cells undergo metabolic reprogramming driven primarily by HIF. HIF regulates the expression of numerous genes involved in metabolic reprogramming, including those involved in glucose metabolism, glycolysis, and mitochondrial function. Additionally, hypoxia fosters the accumulation of reactive oxygen species within cells, potentially contributing to oxidative stress and further amplifying metabolic reprogramming. In conclusion, hypoxia and metabolic reprogramming synergistically facilitate tumor immune evasion (Fig. **3**).

## 8 EPIGENETICS

**Fig. (4):**
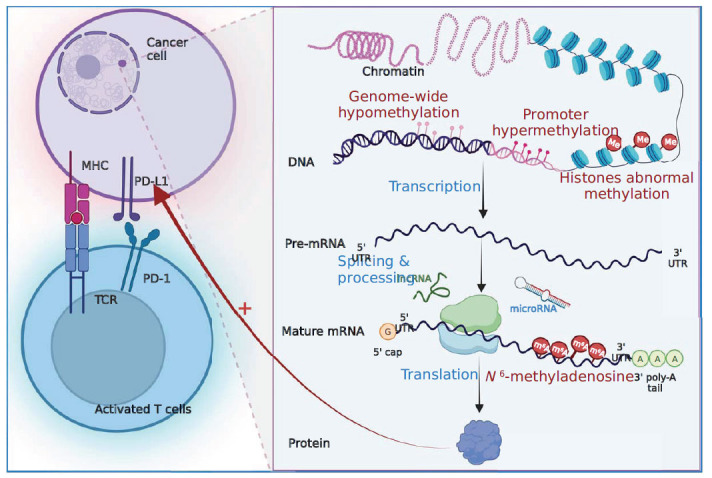
Mechanisms of drug resistance associated with epigenetics.

Epigenetics pertains to the regulation of heritable alterations in gene expression during cell division without modifying the DNA sequence [144]. Key areas of investigation in epigenetics include DNA/RNA methylation, histone modifications, and non-coding RNAs [145]. Perturbations in these processes result in aberrant gene expression and contribute significantly to the initiation and progression of digestive tumors (Fig. **4**).

### 8.1 DNA/RNA Methylation

DNA methylation is a prominent epigenetic modification involved in the regulation of gene expression; the addition of a methyl group to cytosine on the CpG sequence within a gene's promoter region, mediated by DNA methyltransferase, leads to gene silencing. Promoter hypermethylation and genome-wide hypomethylation are the hallmark methylation changes in cancer [146]. In the MSI-H CRC, methylation of the HLA-A promoter results in reduced expression of the HLA-ABC gene, impairing antigen presentation and contributing to drug resistance [52]. Similarly, DNA methylation in HCC can modulate genes associated with immune surveillance [147]. Furthermore, non-squamous gastrointestinal tumors (CRC, GC, CHOL, and HCC) exhibit a distinct immune 'cold' tumor phenotype and drug resistance when characterized by high alternate promoter burden [148, 149]. In GC, PD-L1 expression is increased through various mechanisms, including gene amplification, utilization of ectopic promoters [150], and disruption of structural variants in the 3' region of the PD-L1 gene. Notably, in the presence of secreting T cells, cells with a disrupted 3'- untranslated region (UTR) can more effectively upregulate PD-L1 expression, thereby evading anti-tumor immune responses [151]. The dysregulation of epigenetic modifications in RNA contributes to the development of various cancers. Among these, N6-methyladenosine (m6A) is a prevalent internal RNA modification at the post-transcriptional level [152]. Emerging evidence has demonstrated the indispensable role of m6A methylation in the remodeling of the TIME and in regulating immune surveillance. Importantly, m6A modification plays a critical role in promoting tumor immune escape by modulating key processes, such as hypoxia, metabolic reprogramming, acidosis, and immune cell dynamics within the TME.

### 8.2 Histone Modifications

Histone methylation is a prominent histone modification that is involved in the regulation of gene transcription. Specifically, H3K4me3 is a critical histone modification that preferentially localizes to gene promoters and transcription initiation sites, facilitating the activation of target gene transcription [153]. In the mouse PC, H3K4me3 has been implicated in promoting pancreatic tumor growth and progression through the activation of the osteopontin (OPN)-CD44 axis, which in turn promotes immune evasion in pancreatic cancer [154]. In HCC, lysine-specific demethylase 1A (LSD1), the first identified histone-specific demethylase, has been found to enhance the immunosuppressive activity of HCC by promoting PD-L1 abundance and reducing the methylation of myocyte enhancer factor 2D (MEF2D) [155]. Moreover, overexpression of histone deacetylases (HDACs) has been associated with tumor immune evasion, as observed in both GC [156] and PDAC [157]. This mechanism involves a positive correlation between high HDAC expression and increased PD-L1 expression [158]. Collectively, these findings highlight the critical role of histone modifications, including methylation and deacetylation, in modulating immune responses and promoting immune evasion in various cancers.

### 8.3 Non-coding RNAs

Non-coding RNAs (ncRNAs) are a class of transcriptional mediators that undergo post-transcriptional splicing and do not encode proteins. Instead, they exert tissue-specific pro-tumorigenic or anti-tumorigenic effects [159]. The functional repertoire of ncRNAs includes microRNAs (miRNAs), long ncRNAs (lncRNAs), and circular RNAs (circRNAs). miRNAs are a class of endogenous, short, noncoding, single-stranded RNAs that play significant regulatory roles in cancer biology. In particular, miRNAs have emerged as key regulators of PD-L1 expression through direct binding to the 3′ UTR of the PD-L1 gene. Genomic alterations affecting the PD-L1 3′ UTR region are frequently observed in gastric adenocarcinoma, and several miRNAs have been identified as direct controllers of PD-L1 expression by interacting with its 3′ UTR [151, 160, 161]. Among these miRNAs, miR-105-5p has been shown to modulate the immunogenicity of cancer cells and enhance the activation of CD8+T cells [162]. Aberrant expression of miR-105-5p has been documented in various cancer types, suggesting that dysregulation of miRNAs could serve as a potential mechanism underlying drug resistance. lncRNAs are a class of non-coding RNAs that are more than 200 nucleotides long and exhibit aberrant expression patterns in tumor tissues and cell lines. Through various mechanisms involving transcriptional and translational regulation, lncRNAs exert their effects by modulating the activity of specific genes, either by activating or repressing their expression. In CRC, epigenetically induced lncRNA1 (EPIC1) has been identified as a key player in tumorigenesis. EPIC1 functions by recruiting the histone methyltransferase EZH2, thereby leading to the epigenetic silencing of the IFN-γ receptor and antigen-presenting genes in tumor cells [163]. Notably, this newly discovered mechanism contributes to tumor immune evasion, providing novel insights into the intricate interplay between lncRNAs and immune modulation in CRC. circRNAs are a distinct class of non-coding RNAs that are formed through a process known as the reverse splicing of mRNA precursors and possess a closed-loop structure. Unlike mRNA, circRNAs lack a 5'-end cap and a 3'-end polyA tail [164]. In GC, circRNA discs large MAGUK scaffold protein 1 (circDLG1) has been identified as a contributor to tumor progression. Functionally, circDLG1 enhances the infiltration of MDSCs, thereby promoting tumor growth and metastasis. Mechanistically, circDLG1 interacts with miR-141-3p, which ultimately results in increased expression of the chemokine ligand C-X-C motif chemokine ligand 12 (CXCL12), a chemokine associated with tumor immune evasion and therapy resistance, including resistance to anti-PD-1-immunotherapy [165].

Collectively, the interplay between epigenetic modifications and the immune microenvironment plays a crucial role in regulating tumor initiation, progression, and therapeutic outcomes in cancer. Understanding the complex crosstalk between epigenetics and the immune microenvironment has significant implications for elucidating the mechanisms underlying cancer pathogenesis and for developing innovative therapeutic strategies targeting both epigenetic dysregulation and immune modulation.

## 9 THERAPEUTIC STRATEGIES TO OVERCOME DRUG RESISTANCE IN ONCOLOGY TREATMENT

The important and main findings of the study should come first in the Results Section. The tables, figures and references should be given in sequence to emphasize the important information or observations related to the research. The repetition of data in tables and figures should be avoided. Results should be precise.

### 9.1 Immunonormalisation Therapy for Cancer

Tumors employ various immune evasion mechanisms, necessitating the identification of these mechanisms to improve cancer immunotherapy. Previous immunotherapies aimed to activate or modulate generic control mechanisms to enhance the immune response against the tumor, but this approach, known as “boosted immunotherapy,” often resulted in frequent adverse reactions [166]. In contrast, research now suggests that cancer actively employs diverse strategies to alter or block anti-tumor immunity, known as “immune escape” [167]. By targeting these mechanisms of immune escape in the TME, lost anti-tumor immunity can be restored through “cancer immune normalization therapies,” such as anti-PD therapy. The concept of immune normalization underscores the importance of identifying and correcting specific deficiencies or dysfunctions in the immune response during tumor progression to restore natural antitumor immunity [168]. Currently, numerous therapeutic approaches combining different strategies aimed at addressing tumor immune escape mechanisms are showing promising results.

## 10 COMBINATION THERAPY

### 10.1 Combination Therapy with Other ICIs

Combination therapy targeting PD-1 and CTLA4 has emerged as the most potent approach for treating various malignancies [169]. However, this combination therapy is also associated with a higher frequency of immune-related adverse events. In addition to CTLA-4, the combined inhibition of PD-1 and other immune checkpoints, such as TIM3 [170, 171], LAG3 [172], B7 superfamily member 1 (B7S1) [31] and TIGIT) [173-176] holds promising potential as a therapeutic strategy in digestive malignancies.

### 10.2 Combination Therapy with Chemotherapy

The combination of anti-PD therapy and chemotherapy has exhibited promising outcomes for gastrointestinal tumors. In the resectable ESCC, tislelizumab combined with chemotherapy as neoadjuvant therapy has demonstrated encouraging anti-tumor activity, resulting in high rates of major pathological response (MPR), pathological complete response (pCR), and R0 resection while maintaining acceptable tolerability [177]. Similarly, a randomized phase II clinical trial in locally advanced ESCC revealed improved efficacy with a combination of socazolimab, nab-paclitaxel, and cisplatin [178]. Notably, compared to monotherapy, the use of nivolumab in addition to other chemotherapeutic agents in patients with advanced GC, gastro-oesophageal junction cancer (GEJC), or esophageal adenocarcinoma (EAC) has shown superior OS, PFS, and an acceptable safety profile [179]. Furthermore, serplulimab, in combination with other chemotherapeutic agents, significantly enhanced PFS and OS in previously untreated PD-L1 positive advanced ESCC patients while maintaining a manageable safety profile [180]. These findings highlighted the potential of combining anti-PD therapy with chemotherapy as a promising treatment strategy for gastrointestinal malignancies.

## 11 COMBINATION THERAPY WITH TARGETED THERAPY

### 11.1 Combination Therapy with Molecularly Targeted Drugs

The combination of anti-PD therapy with molecular-targeted drugs has emerged as a promising approach. Notably, in unresectable HCC cases, the use of lenvatinib in conjunction with PD-1 inhibitors has yielded prolonged patient survival, along with a significant ORR and disease control rate (DCR) [181], and substantial improvements in survival outcomes in patients with advanced HCC [182]. Furthermore, a randomized multicenter phase II study revealed the effectiveness of combining atezolizumab with cobimetinib in patients with advanced cholangiocarcinoma (CCA) who had previously undergone first-or second-line therapy [183]. These findings underscore the potential of integrating anti-PD therapy with molecular-targeted agents as a valuable treatment strategy.

### 11.2 Combination Therapy with Targeted Epigenetics

As previously discussed, alterations in histone modifications and DNA methylation within tumor cells have a profound impact on shaping the cancer immune landscape. Thus, it is crucial to address tumor immune evasion from an epigenetic perspective. Studies have revealed that Pretreatment of CD8+T cells with decitabine, a low-dose DNA demethylating agent, leads to enhanced proliferation and effector functions when combined with *in vitro* anti-PD-1 treatment [184]. In a metastatic PC model, the combination of entinostat and anti-PD-1, anti-CTLA-4, or a dual combination significantly improved tumor-free survival [185]. Moreover, miR-329-3p inhibited PD-L1 expression by targeting KDM1A in HCC, thereby augmenting HCC cell responsiveness to the cytotoxic effects induced by T cells [155]. Similarly, in esophageal cancer, the coadministration of the DNA methyltransferase inhibitor 5-Aza and a PD-L1 inhibitor enhanced tumor cell recognition by CTLs [186]. In conclusion, targeting epigenetic modifiers in conjunction with ICIs has the potential to enhance immunogenicity, remodel effective T-cell function, and modify the immunosuppressive TME.

### 11.3 Combination Therapy with Targeted Microbiota

The richness and diversity of the gut microbiota have emerged as influential factors in determining the clinical response to anti-PD therapy, underscoring the potential of combination therapy. Fecal microbiota transplantation (FMT) is a therapeutic intervention in which stool from a healthy donor is administered to the recipient's gastrointestinal tract either *via* colonoscopy or orally, with the primary objective of restoring the balance and functionality of the intestinal microbiota to address disease. FMT has shown promising outcomes in augmenting the anti-tumor effects of ICIs and surmounting resistance to immunotherapy [187-189]. Nonetheless, the role of FMT in gastrointestinal tumor treatment remains understudied and warrants further investigation. Furthermore, there are other combined strategies targeting the microbiota to foster immune activation and enhance the cancer response to ICIs [190]. Continued research in this domain is imperative to validate these findings and optimize the utilization of microbiota-targeting approaches in ICIs therapy.

In addition, dietary habit alterations can influence tumor development and drug resistance through diverse pathways. One particularly promising methodology in this regard is Molecular Pathological Epidemiology (MPE). Given that malignant and precancerous lesions of the gastrointestinal tract are accessible *via* endoscopy, they serve as practical models for *in vivo* MPE studies. MPE, an emerging discipline, refines pathology into a pathobiological data science. This approach extends beyond evaluating well-defined clinical outcomes, offering predictions for intermediate biomarkers indicative of full-blown diseases [191]. Evidence suggests that MPE plays a crucial role in delineating the impact of diet on tumor pathogenesis *via* microorganisms [192]. Thus, integrating MPE with both *in vivo* and *in vitro* experiments can enhance our comprehension of disease pathogenesis, paving the way for improved therapeutic solutions.

### 11.4 Combination Therapy with Targeted Metabolism

Metabolic reprogramming exerts multifaceted effects on the tumor's immune escape process, making targeting metabolism a promising avenue for intervention. It has been shown that IFNα reprograms glucose metabolism within the HCC tumor microenvironment, thereby liberating T-cell cytotoxic capacities and potentiating the PD-1 blockade-induced immune response [193]. Inhibition of solute carrier family 4 member 4 (SLC4A4) in PDAC cells effectively mitigates TME acidosis and reduces lactate production through glycolysis. Moreover, SLC4A4 targeting enhanced T cell-mediated immune responses, and disrupted macrophage-mediated immunosuppression. Notably, the combination of SLC4A4 inhibition with ICIs demonstrates the ability to overcome immunotherapy resistance and extend patient survival. These findings underscore the potential of SLC4A4 as a therapeutic target and emphasize its synergistic effects with the immune checkpoint blockade in PDAC treatment [194].

In addition, there are many preclinical studies in digestive system neoplasms that have identified a potential combination of anti-PD-1 and targeted therapy (Table **2**) [195-206]. And a number of ongoing and upcoming clinical trials investigating combination therapies (Table **3**).

## CONCLUSION

Digestive system tumors exhibit significant heterogeneity, complicating the comprehension of drug resistance. Given this variability, addressing the intricacies of such tumors is essential for overcoming drug resistance challenges. This review dissects the tumor microenvironment and unique characteristics of digestive system tumors, elucidating drug resistance mechanisms that span immune-mediated resistance, microbial interplay, metabolic processes, and epigenetics. Additionally, we synthesize current combination therapies, including dual ICIs, ICIs with chemotherapy, and ICIs alongside targeted interventions. Though these resistance mechanisms can be analyzed from varied perspectives, their complexity and interconnected nature persist. In-depth research is requisite for a nuanced understanding. The evolution of PD-1/PD-L1 blockade therapies presents both opportunities and challenges; as therapeutic efficacy augments, concurrent drug resistance issues arise. Future research should focus on minimizing drug resistance, reducing immune-related side effects, and enhancing immunotherapy effectiveness.

## Figures and Tables

**Table 1:** The application of anti-PD therapy in digestive system neoplasms.

**Cancer**	**Systemic Therapy**	**Treatment Options**	**Biomarker**	**Remarks**
Unresectable Locally a/r/m Esophagus and EGJ adenocarcinomas	First-line therapy	Fluoropyrimidine(fluorouracil or capecitabine)+ Oxaliplatin/cisplatin+trastuzumab+pembrolizumab (category 2A)	HER2(+++)	a
-	-	Fluoropyrimidine(fluorouracil or capecitabine) +oxaliplatin+nivolumab (category 1 for PD-L1 CPS ≥ 5; category 2B for PD-L1 CPS <5)	HER2(0) /HER2(+)	a
-	-	Fluoropyrimidine(fluorouracil or capecitabine) +Oxaliplatin+pembrolizumab (category 2A for PD-L1 CPS ≥ 10; category 2B for PD-L1 CPS <10)	HER2(0) /HER2(+)	a
-	-	Fluoropyrimidine(fluorouracil or capecitabine) +cisplatin+pembrolizumab (category 1 for PD-L1 CPS ≥ 10; category 2B for PD-L1 CPS <10)	HER2(0) /HER2(+)	a
-	Second-line/ subsequent therapy	Pembrolizumab/Dostarlimab-gxly(category 2A)	MSI-H/dMMR	a
-	-	Pembrolizumab(category 2A)	TMB-H	a
Unresectable Locally a/r/m Esophagus and EGJ Squamous cell carcinoma	First-line therapy	Fluoropyrimidine(fluorouracil or capecitabine) +oxaliplatin+Nivolumab/nivolumab(category 2A)	-	a
-	-	Fluoropyrimidine(fluorouracil or capecitabine) +Oxaliplatin/Cisplatin+pembrolizumab (category 2A for PD-L1 CPS ≥ 10; category 2B for PD-L1 CPS <10)	-	a
-	-	Nivolumab+ipilimumab(category 2A)	-	a
-	Second-line/ subsequent therapy	Nivolumab(category 1)	-	a
-	-	Pembrolizumab (category 1 for PD-L1 CPS of ≥10)	-	a
-	-	Pembrolizumab/Dostarlimab-gxly(category 2A)	MSI-H/dMMR	a
-	-	Pembrolizumab(category 2A)	TMB-H	a
Unresectable Locally a/r/mGC	First-line therapy	Fluoropyrimidine(fluorouracil or capecitabine) +Oxaliplatin/Cisplatin+Trastuzumabe+pembrolizumab (category 2A)	HER2(+++)	a
-	-	Fluoropyrimidine(fluorouracil or capecitabine) +Oxaliplatin+Nivolumab (category 1 for PD-L1 CPS ≥5;category 2B for PD-L1 CPS <5)	HER2(0) /HER2(+)	a
-	Second-line/ subsequent therapy	Pembrolizumab/Dostarlimab-gxly(category 2A)	MSI-H/dMMR	a
-	-	Pembrolizumab(category 2A)	TMB-H	a
Locally unresectable or medically inoperable nonmetastatic CRC	First-line therapy	Nivolumab/Pembrolizumab/Dostarlimab-gxly(category 2B)	MSI-H/dMMR	b
mCRC	First-line therapy	Pembrolizumab/nivolumab/dostarlimab-gxly/Pembrolizumab+ipilimumab/dostarlimab-gxly+ipilimumab (category 2A)	dMMR/MSI-H	bc
-	-	Nivolumab+ipilimumab(category 2A)	dMMR/MSI-H	bd
-	-	Nivolumab+ipilimumab(category 2B)	dMMR/MSI-H	be
-	Second-line/subsequent therapy	Pembrolizumab/nivolumab/dostarlimab-gxly/nivolumab+ipilimumab(category 2A)	dMMR/MSI-H	b
Metastatic Anal Carcinoma	Subsequent-line therapy	Nivolumab/Pembrolizumab(category 2A)	-	b
a/mSBA	First-line therapy	Nivolumab/pembrolizumab/dostarlimab-gxly/ Nivolumab+Ipilimumab (category 2A)	dMMR/MSI-H	bf
-	First-line/subsequent therapy	Nivolumab/pembrolizumab/Nivolumab+ipilimumab (category 2A)	dMMR/MSI-H	bc
-	Subsequent-line therapy	dostarlimab-gxly(category 2A)	dMMR/MSI-H	be
HCC	First-line therapy	Pembrolizumabb (category 2B)	-	-
-	-	Nivolumab (Child-Pugh Class B only) (category 2A)	-	-
-	-	Nivolumab+ipilimumab (category 2B)	TMB-H	-
-	Subsequent-line therapy	Nivolumab + ipilimumab (Child-Pugh Class A only) (category 2A)	-	b
-	-	Pembrolizumab (Child-Pugh Class A only) (category 2A)	with or without MSI-H	b
-	-	Nivolumab (Child-Pugh Class B only) (category 2A)	-	b
-	-	Nivolumab+Ipilimumab(category 2B)	TMB-H	bg
a/rHCC	Subsequent-line therapy	Dostarlimab-gxly(category 2B)	MSI-H/dMMR	b
Unresectable mBTC	First-line therapy	Pembrolizumab(category 2A)	MSI-H/dMMR	-
-	-	Nivolumab+ipilimumab (category 2B)	TMB-H	-
a/r BTC	Subsequent-line therapy	Dostarlimab-gxly(category 2A)	MSI-H/dMMR	b
BTC	Subsequent-line therapy	Pembrolizumab(category 2A)	MSI-H/dMMR, or TMB-H	b
-	-	Nivolumab+ipilimumab(category 2A)	TMB-H	bg
mPAAD	First-line therapy	Pembrolizumab(category 2A)	MSI-H/dMMR, or TMB-H	h
Locally a/r/mPDDA	Subsequent therapy	Pembrolizumab(category 2A)	MSI-H/dMMR, or TMB-H	h

**Note:** HER2(+++): HER2 overexpression positive, HER2(0)/HER2(+): HER2 overexpression negative, TMB-H: TMB high (≥10 mutations/megabase). Remarks: a: If no prior tumor progression while on therapy with a checkpoint inhibitor, b: If no previous treatment with a checkpoint inhibitor, c: whether or not intensive therapy, d: With intensive therapy, e: no intensive therapy, f: Patient with prior oxaliplatin exposure in the adjuvant setting or contraindication, g: For patients with disease refractory to standard therapies or who have no standard treatment options available, h: Good/Poor PS (performance status).

**Table 2:** Preclinical studies of the combination of anti-PD-1 and targeted therapy in digestive system neoplasms.

**Cancer Types**	**Target(s)**	**Combined Therapy**	**Mechanisms**	**References**
DGC	uPAR	anti-uPAR monoclonal antibody+ Anti-PD1 Ab	Inhibits tumor growth and prolongs survival	[195]
DGC	PDGFC	PDGFRα/β blockade+ Anti-PD1 Ab	(1) Reversed the immunosuppressive microenvironment through stromal modification;(2) suppressed the growth of fibrotic tumors	[196]
Esophageal carcinoma	MAGE-A11	5-aza-2'-deoxycytidine+ Anti-PD1 /PD-L1 Ab	5-aza-2'-deoxycytidine increased MAGE-A11 expression and subsequently enhanced the cytotoxicity of MAGE-A11-specific CD8+T cells against cancer cell lines	[186]
CRC	VEGFR	Fruquintinib + Anti-PD-1 Ab	(1) Promote release of chemotactic factors; (2) increase CD8+ T cell recruitment and activation; (3) decrease ratio of Treg cell; (4) raise M1/M2 ratio in macrophages	[197]
CRC	VEGFR	Regorafenib+ Anti-PD1 Ab	(1) Enhance M1 macrophage differentiation and activation; (2) continuously inhibit Treg cell infiltration	[198]
CRC	RTKs	Foretinib + Anti-PD1 Ab	(1) Increase PD-L1 level by activating JAK2-STAT1 pathway; (2) further improve T cells' infiltration and function; (3) lower the proportion of TAMs and inhibit polarization to M2	[199]
CRC	PI3K	QA + Anti-PD1 Ab	Enhance tumor regression mediated by cytotoxic T cells	[200]
CRC	TGFβ	SAR439459 + Anti-PD1 Ab	(1) Augment CD8+ T cells' proliferation and inhibit them from exhausted; (2) promote anti-tumor responses of CD8+ T cell; (3) evoke cytokines that are proinflammatory	[201]
CRC	HPK	CompK + Anti-PD1 Ab	(1) Enhance T-cell immune responses; (2) increase sensitivity of TCR to recognize TAAs	[202]
CRC	Epigenetic	Decitabine+Anti-PD1 Ab	(1) Enhances antitumor response in multiple tumor models and significantly promotes the activation and expansion of CD8 progenitor Tex; (2) sustains the expression levels of JunD and target genes in CD8 T cells from solid tumor models	[184]
aICC	METTL1	METTL1 depletion /CXCR2 inhibition+ +Anti-PD1 Ab	(1) Inhibits the accumulation of PMN-MDSCs in ICC tumors; (2) Inhibits the translation rate of CXCL8; (3) Restore T cell proliferation and the anti-tumoral activity	[203]
HCC CRC	VEGFR	Lenvatinib + Anti-PD1 Ab	Activate CD8+ T cells *via* reducing TAMs and activating the interferon pathway	[204]
HCC	Exosomal circCCAR1 or CCAR1	Exosomal circCCAR1 or CCAR1 inhibitor+Anti-PD1 Ab	(1) Inhibition of the ircCCAR1/miR-127-5p/WTAP positive feedback loop; (2) attenuation of PD1 protein stability and prevention of CD8+ T cell dysfunction; (3) reduction of PD-L1 transcription to prevent drug resistance	[205]
HCC	TIM3/LAG3	Anti-TIM-3/anti-LAG-3/anti-CTLA-4 Ab +Anti-PD1 Ab	Enhanced the functionality of CD4 or CD8 TILs	[172]
HCC	KDM1A mRNA	miR-329-3p	(1) Inhibits tumor cellular immunosuppression; (2) reinforces the response of tumor cells to T cell-induced cytotoxic effect by targeting KDM1A mRNA and downregulating its expression; (3) contributes to MEF2D demethylation and activation of PD-L1 expression	[155]
HCC	Glucose metabolism	IFNα+Anti-PD1 Ab	(1) IFNα corrected tumor-imposed glucose competition in the HCC microenvironment; (2) restored the effector function of CD8+ T cells by increasing CD27 transcription.	[193]
HCC	ZFP64	Gö6976/ Lenvatinib + Anti-PD1 Ab	Reset the tumor microenvironment and restore sensitivity to anti-PD1 by blocking the PKCα/ZFP64/CSF1 axis.	[206]
PDAC	Epigenetic	Entinostat (ENT)+anti-PD-1+anti-CTLA-4	Altered infiltration and function of innate immune cells	[185]
PDAC	SLC4A4	SLC4A4 inhibitor Anti-PD-1 anti-CTLA-4	(1) Mitigate tumor acidosis; (2) abate immunosuppression; (3) increase CD8 T cell fitness; (4) sensitize PDAC to the current immunotherapeutic regimens	[194]
PDAC	TIGIT	Anti- TIGIT Ab + anti-PD-1 Ab	Significantly enhanced the proliferation of CD8 TILs	[176]
PDAC	TIGIT	Anti-TIGIT Ab + anti-PD-1 Ab + CD40a	(1) Tumor-reactive (neoantigen-specific) CD8 TILs express high levels of TIGIT;(2) Agonistic CD40 antibodies bypass the need for CD4 T cell help and can enhance anti-tumor responses with ICB	[173]

**Abbreviations:** FGF: fibroblast growth factor; PI3K: phosphoinositide 3-kinase; RTKs: receptor tyrosine kinase; IFNγ: interferon γ; QA: (-)-4-O-(4-O-β-D-glucopyranosylcaffeoyl) Quinic Acid, SNA: (S)-(-)-N-[2-(3-Hydroxy-1H-indol-3-yl)-methyl]-acetamide), TAAs: tumor-associated antigens, METTL1: m7G tRNA methyltransferase, CCAR1: Cell Division Cycle And Apoptosis Regulator 1, Tex: CD8+ exhausted T cells, MAGE-A11:Melanoma-associated antigen A11, EC: esophageal cancer, CD40a: CD40 agonist, DGC: diffuse-type gastric cancer, uPAR: urokinase-type plasminogen activator receptor, PDGFC: Platelet-derived growth factor C, ZFP64: zinc finger protein 64.

**Table 3:** Ongoing and upcoming clinical trials investigating combination therapies in digestive system neoplasms.

**NCT Number**	**Phase**	**Cancer Types**	**Anti-PD-1/PD-L1**	**Combined Therapy**	**Targets**	**Estimated Sample Size**	**Status**	**Primary Outcome Measures**
NCT02999295	I/II	GC/GEJC	Nivolumab	Ramucirumab	VEGFR	46	Completed	DLTs
NCT04879368	III	GC	Nivolumab	Regorafenib	VEGFR	450	Recruiting	OS
NCT04503967	II	GC	Nivolumab	Anlotinib	VEGFR	48	Not yet recruiting	ORR
NCT03609359	II	aGC	Pembrolizumab	Lenvatinib	VEGFR	29	Completed	ORR
NCT05041153	I	aGC	Pembrolizumab	Lenvatinib	VEGFR	15	Not yet recruiting	ORR
NCT04745988	II	GC	Pembrolizumab	Lenvatinib	VEGFR	43	Recruiting	Major pathological response rate
NCT03321630	II	mGC	Pembrolizumab	Lenvatinib	VEGFR	24	Recruiting	ORR
NCT04798781	II	aGC/GEJC	Pembrolizumab	Telatinib	VEGFR	17	Recruiting	Progression-free survival
NCT04164979	II	mGC/GEJC	Pembrolizumab	Cabozantinib	VEGFR	20	Recruiting	Percentage of Participants with Progression-free Survival at 6 Months
NCT02443324	I	GC/GEJC	Pembrolizumab	Ramucirumab	VEGFR-2	298	Active, not recruiting	DLTs
NCT04069273	II	aGC	Pembrolizumab	Ramucirumab	VEGFR-2	58	Recruiting	BORR
NCT04632459	II	mGC	Pembrolizumab	Ramucirumab	VEGFR-2	35	Not yet recruiting	ORR
NCT03797326	II	aGC/CRC	Pembrolizumab	Lenvatinib	VEGFR	590	Active, not recruiting	ORR
NCT03406871	I	aGC/CRC	Nivolumab	Regorafenib	VEGFR	50	Completed	RD of Regorafenib
NCT03995017	I/II	aGC	Nivolumab	Ramucirumab/Rucaparib	VEGFRPARP	34	Recruiting	RP2D, ORR
NCT02901301	III	HER2+ GC	Pembrolizumab	Trastuzumab	HER-2	41	Active, not recruiting	The recommended dose of phase II
NCT02954536	II	HER2+ GC	Pembrolizumab	Trastuzumab	HER-2	37	Active, not recruiting	Percentage of Participants With Progression Free Survival
NCT04249739	II	HER2+ GC	Pembrolizumab	Trastuzumab	HER-2	93	Recruiting	ORR
NCT02689284	I/II	HER2+ GC	Pembrolizumab	Margetuximab	HER-2	95	Recruiting	Determine the recommended expansion phase cohort (Cohort 2) dose of margetuximab
NCT04278144	I/II	HER2+ aGC	Nivolumab	BDC-1001	HER2-TLR7/8	390	Recruiting	AEs, SAEs, DLTs, MTD, ORR
NCT05111626	Ib/III	aGC/GEJC	Nivolumab	Bemarituzumab	FGFR	528	Not yet recruiting	DLTs, TEAEs
NCT02946671	I	GC	Nivolumab	Mogamulizumab	CCR4	16	Completed	Number of patients with adverse events
NCT03044613	I	aGC	Nivolumab	Relatlimab	LAG-3	32	Active, not recruiting	Number of participants with treatment-related adverse events
NCT04062656	II	aGC/GEJC	Nivolumab	Relatlimab	LAG-3	21	Recruiting	Rate of pathological complete responses
NCT04110093	I/II	CRC	Nivolumab	Regorafenib	VEGFR	120	Recruiting	ORR, PFS
NCT04072198	II	CRC	Nivolumab	Bevacizumab	VEGFR	70	Completed	ORR
NCT03377361	I/II	CRC	Nivolumab	Trametinib	VEGFR	232	Recruiting	DLTs, AEs, SAEs, ORR
NCT02298959	I	aCRC	Pembrolizumab	Ziv-aflibercept	VEGFR	78	Recruiting	RD of ziv-aflibercept and pembrolizumab
NCT03657641	I/II	mCRC	Pembrolizumab	Regorafenib	VEGFR	75	Recruiting	DLTs, PFS, OS
NCT05035381	II	MSI-H CRC	Pembrolizumab	Bevacizumab	VEGFR	10	Recruiting	ORR
NCT02713373	I/II	CRC	Pembrolizumab	Cetuximab	EGFR	45	Completed	To Examine the Adverse Event Profile, PFS
NCT04429542	I	aCRC	Pembrolizumab	BCA101	EGFR TGFβ	292	Recruiting	Safety, Tolerability, DLTs
NCT02903914	I/II	aCRC	Pembrolizumab	INCB001158	ARG	260	Active, not recruiting	Determination of the Safety and Tolerability
NCT03239145	I	aCRC	Pembrolizumab	Trebananib	ANG	60	Active, not recruiting	DLT
NCT02060188	II	CRC	Nivolumab	Cobimetinib/BMS-986016	MET/LAG-3	385	Active, not recruiting	ORR
NCT04344795	I	CRC	Pembrolizumab	TPST-1495	EP	175	Recruiting	RP2D
NCT02713529	I/II	CRC	Pembrolizumab	AMG 820	CSF1R	117	Completed	DLT, TEAEs, ORR
NCT03473925	II	aCRC	Pembrolizumab	Navarixin (MK-7123)	CXCR	107	Completed	ORR, DLTs
NCT03332498	I/II	aCRC	Pembrolizumab	Ibrutinib	BTK	40	Active, not recruiting	RP2D
NCT02972034	I	aCRC	Pembrolizumab	MK-8353	ERK1/2	110	Active, not recruiting	AE, DLT
NCT04721301	I	mCRC	Nivolumab	Maraviroc	CCR	50	Active, not recruiting	Number of participants and severity of treatment-related adverse Events
NCT03184870	I/II	aCRC	Nivolumab	BMS-813160	CCR	332	Active, not recruiting	AEs, SAEs, DLT
NCT03374254	I	mCRC	Pembrolizumab	Binimetinib	MEK	220	Active, not recruiting	DLT
NCT03126110	I/II	mCRC	Nivolumab	INCAGN01876	GITR	145	Active, not recruiting	TEAE, ORR
NCT03241173	I/II	mCRC	Nivolumab	INCAGN01949	OX40	52	Completed	TEAE, ORR
NCT04729322	II	dMMR CRC	Pembrolizum/Nivolumab	FMT		15	Recruiting	ORR
NCT03797326	II	CRC/BTC/PC	Pembrolizumab	Lenvatinib	VEGFR	590	Active, not recruiting	ORR
NCT04130763	I	Gastrointestinal	Anti-PD-1	FMT		10	Unknown	ORR
NCT04829383	II	HCC	Atezolizumab	Bevacizumab	VEGF	50	Recruiting	Frequency and severity of toxicities
NCT04770896	II	HCC	Atezolizumab	Lenvatinib	TKI	554	Recruiting	OS
NCT04826406	III	HCC	Camrelizumab	Apatinib	TKI	40	Recruiting	ORR
NCT04443309	I/II	HCC	Camrelizumab	Lenvatinib	TKI	53	Recruiting	ORR
NCT05048017	II	HCC	Camrelizumab	Regorafenib	TKI	20	Recruiting	PFS
NCT04806243	II	HCC	Camrelizumab	Regorafenib	TKI	69	Recruiting	OS
NCT04310709	II	HCC	Nivolumab	Regorafenib	TKI	42	Recruiting	ORR
NCT04170556	I/II	HCC	Nivolumab	Regorafenib	TKI	78	Recruiting	Rate of AE, related AEs, death
NCT04039607	III	HCC	Nivolumab	Ipilimumab	CTLA-4	728	Recruiting	OS
NCT05199285	II	HCC	Nivolumab	Ipilimumab	CTLA-4	40	Not yet recruiting	ORR
NCT02777710	I	PC/CR	Durvalumab	Pexidartinib+Durvalumab	CSF1-R Flt3 Kit	48	Completed	DLT、ORR
NCT05327582	I/II	PC/BTC	Durvalumab	Durvalumab+Lenvatinib+Nab paclitaxel	TKI	65	Recruiting	AEs, ORR
NCT05431270	I	PC	Anti-PD-1 antibody	PT199+Anti-PD-1 antibody	CD73	41	Recruiting	MTD
NCT04060342	I	mPC	Pembrolizumab	GB1275+nab-paclitaxel and gemcitabine+ pembrolizumab	CD11b	61	Terminated	DLTs, AEs, ORR

**Abbreviations:** ORR: Objective response rate, BORR: best overall response rate, RD: Recommended Dose of Regorafenib, MPR: Major pathological response rate, DLTs: Dose-Limiting Toxicities, MTD: Maximum tolerated dose, TEAEs: Treatment -Emergent Adverse Events, RP2D: Phase I - Recommended Phase II Dose, AE: Adverse Event, SAEs: serious adverse events, DCR: Disease Control Rate, MEK: mitogen-activated protein kinase, TGFβ: transforming growth factor-β, MEK: mitogen-activated protein kinase, CXCR: chemokine receptor, CSF1R colony-stimulating factor 1 receptor, BTK: Bruton tyrosine kinase, ARG: arginase, ERK: extracellular signal-regulated kinase, ANG: angiotensin, EP: E-type prostate receptor, CCR: chemokines receptor, PARP: poly ADP-ribose polymerase, FGFR: fibroblast growth factor receptor, TLR: toll-like receptor, MET: mesenchymal-epithelial transition factor, RANKL: receptor activator of nuclear factor-κ B ligand, GITR: glucocorticoid-induced tumor necrosis factor receptor, OX40: Tnfrsf4.

## LIST OF ABBREVIATIONS

2HG: 2-hydroxyglutarate
Ag: Antigen
APCs: Antigen-presenting Cells
ATB: Antibiotics
β2m: β2- microglobulin
B7S1: B7 Superfamily Member 1
CAFs: Cancer-associated Fibroblasts
CCA: Cholangiocarcinoma
CHOL: Cholangiocarcinoma
circDLG1: circRNA Discs Large MAGUK Scaffold Protein 1
circRNAs: Circular RNAs
CRC: Colorectal Cancer
CTLA-4: Cytotoxic T-lymphocyte Antigen 4
CTLs: Cytotoxic T Lymphocytes
CXCL12: C-X-C Motif Chemokine Ligand 12
DCR: Disease Control Rate
DCs: Dendritic Cells
dMMR/MSI-H: Deficient DNA Mismatch Repair/Microsatellite Instability-high
EAC: Esophageal Adenocarcinoma
EC: Esophageal Cancer
ECM: Extracellular Matrix
ENT1/2: Equilibrative Nucleoside Transporter 1 or 2
EPIC1: Epigenetically Induced lncRNA 1
ESCC: Esophageal Squamous Carcinoma
EZH2: Enhancer of Zeste Homolog 2
FAs: Fatty Acids
FMT: Fecal Microbiota Transplantation
FOXP3: Forkhead Box P3
GC: Gastric Cancer
GEJC: Gastro-oesophageal Junction Cancer
GI: Gastrointestinal
HCC: Hepatocellular Carcinoma
HDACs: Histone Deacetylases
HIF: Hypoxia-inducible Factor
HLA: Human Leukocyte Antigen
ICC: Intrahepatic Cholangiocarcinoma
ICIs: Immune Checkpoint Inhibitors
LAG3: Lymphocyte-activating 3
lncRNAs: Long ncRNAs
LSD1: Lysine-specific Demethylase 1A
LTA: Lipoteichoic Acid
m6A: N6-methyladenosine
mAbs: Monoclonal Antibodies
MAMPS: Microbial-associated Molecular Patterns
MCT4: Monocarboxylate Transporter 4
MDSCs: Myeloid-derived Suppressor Cells
MEF2D: Myocyte Enhancer Factor 2D
MHC: Major Histocompatibility Complex
miRNAs: microRNAs
MPE: Molecular Pathological Epidemiology
MPR: Major Pathological Response
MSS: Microsatellite Stable
ncRNAs: Non-coding RNAs
OPN: Osteopontin
OS: Overall Survival
PC: Pancreatic Cancer
pCR: Pathological Complete Response
PDAC: Pancreatic Ductal Adenocarcinoma
PFS: Progression-free Survival
PIK3CA: Phosphatidylinositol-4,5-bisphosphate 3- kinase Catalytic Subunit Alpha
pMMR: Proficient Mismatch Repair
PTEN: Phosphatase and Tensin Homolog
SCFAs: Short-chain Fatty Acids
SLC4A4: Solute Carrier Family 4 Member 4
TAMs: Tumor Associated Macrophages
TCR: T-cell Receptor
TIGIT: T-cell Immunoglobulin and ITIM Domain
TILs: Tumor-infiltrating Lymphocytes
TIM3: T Cell Immunoglobulin and Mucin-Domain Containing-3
TIME: Tumor Immune Microenvironment
TMB: Tumor Mutational Load
TME: Tumor Microenvironment
Tregs: Regulatory T Cells
UTR: Untranslated Region
VEGF: Vascular Endothelial Growth Factor
